# Editorial: Cooperation of gene regulatory networks and phytohormones in cell development and morphogenesis

**DOI:** 10.3389/fpls.2023.1290538

**Published:** 2023-10-04

**Authors:** Muhammad Asim, Yan Zhang, Wenwen Liu

**Affiliations:** ^1^ Key Laboratory of Tobacco Biology and Processing, Ministry of Agriculture and Rural Affairs, Tobacco Research Institute, Chinese Academy of Agricultural Sciences, Qingdao, China; ^2^ National Key Laboratory of Plant Molecular Genetics, CAS Center for Excellence in Molecular Plant Sciences, Institute of Plant Physiology and Ecology, Chinese Academy of Sciences, Shanghai, China; ^3^ CAS Key Laboratory of Biofuels, Shandong Provincial Key Laboratory of Synthetic Biology, Qingdao Institute of Bioenergy and Bioprocess Technology, Chinese Academy of Sciences, Qingdao, Shandong, China

**Keywords:** morphogenesis, phytohormones, cell growth, genes, gene regulatory network (GRNs), transcription factors

The cooperation between gene regulatory networks (GRNs) and phytohormones in cell development and morphogenesis represents a cornerstone of plant developmental biology. The interaction between these two biological systems governs many aspects of plant growth and differentiation, helping to shape the architecture of plants in response to various environmental and internal signals (Van den Broeck et al., 2020). GRNs consist of transcription factors (TFs), target genes, and other regulatory elements that coordinate the expression of genes in response to developmental cues and refer to the complex web of gene interactions that determine when and where individual genes are activated or repressed (Huang and Kauffman, 2009). This Frontiers in Plant Science virtual Research Topic on the “*Cooperation of Gene Regulatory Networks and Phytohormones in Cell Development and Morphogenesis*” consists of five publications, including two reviews and three original research articles.

## 1 Insights into GRNs and phytohormones

Thousands of genes are expressed in a cell and work together to maintain the cell’s fitness, function, and survival. To provide the optimal functional result, each gene must be expressed at the proper time and suitable levels, manipulated by GRNs (Davidson and Levine, 2008). The regulation of genes’ expression is incredibly strong. For instance, the genes involved in cell development in a particular cell type vary very little from one to another; however, some gene expression is highly variable: their levels are noisy and are different from cell to cell and individual to individual. This can greatly benefit physiological responses to outside cues and stresses (Macneil and Walhout, 2011). Phytohormones are organic compounds, and their small amount affects plant growth and development (Davies, 2010). Some of the most well-known phytohormones include auxins, cytokinins, gibberellins, ethylene, abscisic acid, and JA. These hormones can stimulate or inhibit growth, mediate cellular differentiation, and contribute to the plant’s response to environmental stimuli (Vaishnav et al., 2023). It is the cooperation of GRNs and phytohormones that inhibits cell development and morphogenesis.

To briefly outline recent technological progress made to elucidate GRNs in plants, such as the techniques that allow us to characterize physical interactions in GRNs in plants and to analyze their regulatory consequences, combining genome-wide experimental approaches with mathematical modeling allows us to obtain deeper insights into key regulatory interactions and the combinatorial control of important plant processes. About 15 years ago, the availability of high-throughput gene expression data made it possible to predict large-scale GRNs. Many different ways have been explored (Madhamshettiwar et al., 2012). Yao et al. identified the essential genes regulating rhizome axillary bud regrowth in perennial rice by trend analysis of expressed genes at different time periods and co-expression network creation through transcriptome sequencing technology. With the build-up of the connection between biology and other disciplines, modeling these networks is a big challenge that needs to be resolved. (Mishra et al., 2018). Yu et al. employed the “single-pole dual-control (SDPC)” competitive mechanism in plants to explain plant development and microbial infection. Furthermore, gene networks show how groups of genes work together to complete tasks. To comprehend the underlying biological process and its molecular system, targeted manipulation lets us investigate gene regulatory relationships (Kaufmann and Chen, 2017).

The cis-regulatory genomic DNA regions in or around their target genes are directly bound with TFs to regulate gene expression, which can raise or decrease downstream target expression. Other regulators include the transcriptional cofactor that interacts with the TFs physically, chromatin, or components of the basal transcriptional machinery; RNA-binding portions that interact with mRNAs and regulate the translation or mRNA stability; and the mRNAs that inhibit the mRNA stability by hybridizing to the sequence within their mRNA targets (Macneil and Walhout, 2011). GRNs have garnered attention and various statistical inference methods from gene expression data in recent years. Feng et al. found that *Halostachys caspica* pathogenesis-related protein 10 (HcPR10) acts as a cytokinin reservoir because its crystal structure showed a trans-zeatin riboside (cytokinin) deep in its cavity with a conserved conformation and protein–ligand interactions. Despite their popularity, GRNs have been misunderstood, so this current Research Topic studied the GRN more closely, notably by discussing their meaning, their coherence across network inference techniques, ensemble approaches, GRN evaluation, the estimated number of GRNs, and their use in diverse application domains. The discussion has brought greater comprehension of the questions and necessary steps to utilize GRNs in a clinical context and personalize medicine. Through synthetic network biology, such investigations revealed the transcriptional processes that generate strong vs. stochastic gene expression and their effects on phenotypic robustness and variability. Here, we explored the topological features and transcriptional and phenotypic outputs of GRNs in development and organismal physiology.

## 2 Cooperation in cell development

The interaction between GRNs and phytohormones plays a pivotal role in cell development:

### 2.1 Cell division

Hormones such as cytokinins promote cell division, while others such as abscisic acid might inhibit it (El-Showk et al., 2013). The stem cell population in the shoot apical meristem (SAM) is maintained by cytokinin. The two distinct cytokinin-dependent pathways, CLAVATA (CLV)-dependent and CLV-independent, control WUS levels (Gordon et al., 2009). High levels of cytokinin signaling in Arabidopsis cause ectopic *WUSCHEL* (*WUS*) expression and influence neighboring cells’ stem cell fate (Lee et al., 2019). WUS directly represses the transcription of several two-component ARABIDOPSIS RESPONSE REGULATOR genes (ARR5, ARR6, ARR7, and ARR15), which act in the negative-feedback loop of cytokinin signaling (Leibfried et al., 2005). These data imply that cytokinin is adequate for the induction of WUS and the determination of stem cell fate. GRNs determine the expression of genes that are vital for the cell cycle in response to these hormonal signals. As a non-canonical AUXIN RESPONSE FACTOR (ARF), ARF3/ETTIN (ETT) mediates auxin responses to orchestrate multiple developmental processes during the reproductive phase. GRNs respond to hormone cues by regulating cell cycle genes. The ARF3/ETT modulates auxin responses to coordinate reproductive development, according to recent findings by Fu et al. It is stated that ARF3 maintains dynamic SAM non-autonomously. Regarding the temporal aspect, the term “cell differentiation” may also refer to the development of the unicellular organism for the origin of different growth stages and division cycles or the phases in which this cycle is interrupted (Differentiation and Plant, 1964). These antagonistic interactions between CLV and WUS are tightly integrated with hormone function, demonstrating the role of GRNs and phytohormones in cell division regulation.

### 2.2 Cell differentiation

GRNs and phytohormones have a hand in differentiating cells into various tissue types. For instance, the balance between auxin and cytokinin determines whether a cell differentiates into root or shoot tissue. Plants and other eukaryotic use sophisticated gene expression processes to regulate development, environmental response, and cellular homeostasis. Plants generate new organs throughout their lifespan due to the pluripotent stem cell population, unlike mammals. Cell differentiation relies on the TFs, which form the GRNs with the basic cofactors and posttranslational regulators for specialized developmental activities (Kaufmann and Chen, 2017). The activities around DNA are controlled by hormones and chemicals, which decide what is transcribed and what is ignored. The body and nearby cells determine the variables that affect a cell from birth to death (Satterlee et al., 2020). For instance, the pancreas or thyroid may release hormones involved in cellular growth. These TFs directly impact the proteins that transcribe DNA, ultimately producing functioning proteins and more cells. However, when cells congress, they build up communication with one another so there is no more space. Thus, this interaction process between hormones and GRNs on cell differentiation is involved in plant growth and development, including meristem function, vascular development, stress responses, and senescence (Satterlee et al., 2020).

### 2.3 Cell expansion

Hormones such as gibberellins promote cell elongation. GRNs will mediate the expression of genes vital for cellular elongation in response to gibberellin signals. Jasmonic acid (JA) promotes potato tuber formation, reducing the leaf primordial length, meristem enlargement, and cell expansion (Naturales, 2003). Proper root development requires ROS homeostasis, which regulates cell proliferation and differentiation at root tips. Plant roots collect significant quantities of ROS; when this unbalances, the plant cannot adapt to environmental changes and dies. Moreover, ROS controls cell expansion and cell processes such as root hair formation and lateral root development (Mase and Tsukagoshi, 2021). Auxin is involved in cell enlargement and proliferation. During the process of tuberization, auxin in potato plant dynamics affects tuber initiation by stimulating its biomass and increasing tuberization (Aksenova et al., 2012). IAA transport from shoot to root and the stolon is necessary for tuberization (Sergeeva et al., 1994). *StYUC-like 1* expression increases at stolon tips during tuberization (Bachem, 2013). GA targets DELLA degradation in each elongation zone tissue to stimulate root development, which regulates over 100 transcription factors to integrate environmental signals. GA conjugation to GID1, its soluble receptor, increases GID1-GA and DELLA protein interaction, changing their ubiquitin in-proteasome route (Yoshida et al., 2014). GA-GID1-DELLA complexity alters independently of GA presence or absences. Without GA, DELLA conjugates to the protein complex and inhibits the TF, whereas in the presence of GA, GID1 triggers DELLA and TFs (Ariizumi et al., 2008). The DELLA protein may have coordinated multiple developmental programs throughout green lineage evolution by generating co-gene expression in different species. Thus, DELLA recruitment in the gibberellin signaling pathways may have expanded their role in different processes (Briones-moreno et al., 2017).

## 3 Cooperation in morphogenesis

Morphogenesis, the process that gives a plant its shape, is also governed by the interaction of GRNs and phytohormones:

The constant increase in evolution leads to the complexity of the regulatory mechanism and the plants’ developmental process. Throughout their lifetime, plants undergo various morphological and developmental changes. Gene expression is the primary mechanism of development regulation (Kaufmann and Chen, 2017). It is controlled by other regulators that collectively make a network, integrating the environmental constraints and coordinating various developmental programs via GRNs (Kaufmann and Chen, 2017). As previously mentioned, GRNs are a series of regulatory factors that interact among themselves and with other regulators to control the levels of the mRNA and proteins to specify temporal and special patterns (Gene regulatory networks, 2005).

### 3.1 Organ formation

Plant hormone interactions produce complicated regulatory circuits that regulate new meristem growth. They collaborate with GRNs to control genes related to the development of roots, flowers, and fruits (Meitzel et al., 2021). Although many peptides and receptors have been discovered, most are one-way positive signal pathways. Only a few examples regulate the signal routs antagonistically (Lee and De, 2016). However, Yu et al. proposed that antagonistic competitive signaling pathways in plants adopt a “SPDC” competing mode. In plant developmental biology, polarity establishment is the core issue related to organ morphogenesis. Previous research has defined leaf polarity on a three-dimensional spatial axis based on the relative position of the leaf primordium and SAM to examine leaf polarity development (Bowman, 2000). Several studies showed that auxin is the primary regulator of the apical dominant. It is synthesized in the shoot tip and carried down the stem, suppressing the development of the lateral root buds (Kieffer et al., 2010). The regeneration of axillary buds is mainly involved in cell division, tissue differentiation, and organogenesis of the apical meristem (Petra, 2012).

To address these issues, Yao et al. examined axillary bud development trends before and after apical spike removal in perennial rice (PR23) and annual rice (Chugeng28). Annual rice axillary buds at high nodes expanded quicker than others. Through transcriptome sequencing analysis by Yao et al., the PR23 rhizome buds’ global gene expression patterns at compression nodes that developed 1, 3, 4, and 5 days following apical spike removal were evaluated. Moreover, Yao et al. concluded that this research shed light on the differences between the auxiliary bus regeneration pattern of the perennials and annual rice and gave them intricate regulatory networks that are present during the regeneration of rhizome axillary buds in perennial rice. Auxin regulates gene expression by regulating the activity of ARFs via Aux/IAA pathways (Calderón Villalobos et al., 2012). Flowering plants undergo four life stages: embryonic development, vegetative growth, reproductive growth, and senescence. The reproductive growth stage is a crucial period for plant fitness and is of significant importance from the biologists’ and breeders’ points of view (Alvarez-buylla et al., 2010). Genetic and phylogenetic analyses show that most of the *arf* single mutants display no obvious phenotypes, confirming the functional redundancy in the ARF family (Okushima et al., 2005). However, *arf3* mutation disrupts meristem homeostasis, floral determinacy, pattering formation, gynoecium morphogenesis, ovule development, and self-incompatibility (Su et al., 2018).

These findings showed that ARF3 uniquely functions in the auxin signaling and developmental process. To further elaborate on the importance, Fu et al. stated that auxin is involved practically in every aspect of plant growth and development. A recent study showed that ARF3 governs the dynamics of SAM maintenance in a non-cell autonomously. This showed the hierarchical regularity mechanism of ARF3 in the developmental process during the plant’s reproductive process. To specify the primordium density in the principle zone, ARF5 directly activates the auxin transporters PIN FOMRMED 1 (PIN1), the floral meristem identity gene LEAFY (LFY), the organ size regulatory genes AINTEGUMNENTA (ANT) and AINTEGUMENTA-LIKE 6 (AIL6), and the abaxial identity gene FILAMENTOUS FLOWER (FIL) (Wakeel et al., 2018). Furthermore, HcPR10-mediated traits, including bolting, faster flowering, higher branch number, and siliques per plant, are substantially linked with transgenic Arabidopsis cytokinin levels. HcPR10 expression patterns are temporally associated with plant cytokinin levels.

### 3.2 Tissue patterning

The formation of vascular tissues, epidermis, and other plant tissues is controlled by the coordinated action of hormones and GRNs. Leaves are one of the vegetative parts of the plant that are crucial for plant growth and development. The PIN proteins control auxin transport throughout various periods of plant development and have been thoroughly documented in various plant species (Ullah et al., 2021). Chen et al. have examined that *BpPIN3* regulates the expression of the auxin response-related genes and the polar transport of the auxin to alter the polar shape of the proximal and distal axes of the birch leaves. In addition, plant hormones such as ethylene and GAs regulate sex determination in monoecious plants and other factors. In cucumber (*Cucumis statives* L.), GAs are necessary for developing the male flower. Cucumber plants treated with GA and the control plants’ shoot apices were transcriptionally analyzed to give evidence that GA regulates sex determination via both ethylene-dependent and ethylene-independent mechanisms (Zhang et al., 2017).

According to Feng et al., plants create PRs responding to abiotic and biotic stressors. A large intracellular PR-10 family has over 100 members (Xu et al., 2014). PR-10 genes, including reproductive and vegetative tissue, are expressed everywhere in plants, indicating their crucial involvement in growth and development (Liu and Ekramoddoullah, 2018). Furthermore, HcPR10 in *Halostachys caspica* mainly accumulates in vascular tissue, where plant hormones translocate for large distances.

### 3.3 Response to environment

Plant hormones play a crucial role in interpreting environmental signals (detected by receptors) and inducing developmental programs through different interconnected pathways.

Environment signals (light, gravity, microbial infection) may modify the plant’s form. Phytohormones mediate these reactions, and GRNs govern gene expression that supports morphological transformation. Unlike mammals, plants create organs throughout their life cycle, even beyond embryonic development. This is possible because plants possess a small group of pluripotent stem cells in their meristems. The SAM plays a key role in forming all of the aerial structures of plants, including FMs. The FMs subsequently give rise to the floral organs containing reproductive structures.

The study on the model plant Arabidopsis has shown the importance of TFs and secreted peptides in meristem activity. Recent advances in genomic, transcriptomic, imaging, and modeling technologies have allowed us to study the interaction between TFs, secreted peptides, and plant hormones (Lee et al., 2019). Phytotropins, plasmas membrane photoreceptors proteins in flowering plants, perceive blue light and trigger a phototropic response (Sakamoto and Briggs, 2002). Phototropins (PHOT1 and PHOT2) govern the stomatal opening, leaf expansion, stem inhibition, and the blue-light response (Christie, 2007). Another phototropism protein is ROOT PHOTOTROPISM 2 (RPT2). Mutations in the RPT2 cause defective phototropic response under intense blue light (Sakai et al., 2000). Gravity guides major plant structures, including the directional development of the primary root via auxin gradients that are disrupted when the roots depart from the vertical as a gravity sensor (Ferl and Paul, 2016). The vertical toots deliver PIN3/7 proteins evenly to the plasma membrane on all cell sides for the symmetrical auxin distribution to the lateral cap. When the plants are reoriented in the gravity response, these proteins immediately re-localize to the statocytes’ lower sides, forcing auxin to the lower flank of the root caps (Bittner et al., 2022). The transcytosis delocalization of the PIN3 and PIN7 portions to the bottom of the statocytes is induced by gravity. It requires small GTPases of the ADP-ribosylation factor type and GNOM-type GDP/GTP exchange factors (GEFs) (Mohanasundaram and Pandey, 2022).

Auxin regulates plant growth and development, including cell division, elongation, and differentiation, tissue shape, and functions. Despite rosette leaves and other SAM products, auxin biosynthesis, transport, and signaling mutants have bare inflorescences without flowers (Banasiak and Biedro, 2019). Canonical ARF (class A), MONOPTEROS (MP), regulates auxin signaling, such as ARF5 regulating gene expression to determine meristematic and primordium fate signaling (Rademacher and Mo, 2011). Yu et al. claimed that plant growth and pattern formation depend on the diffusible signals and the location cues in this Research Topic, which activates the intracellular downstream components via cell surface receptors that direct cells to adapt specific fates for optimal functions and maintain biological fitness. For instance, Yu et al. proposed that the “SPDC” competitive mode exists in microbial infection. The paracrine and autocrine signaling chemicals compete to bind receptors or receptor complexes, activate antagonistic molecular pathways, and precisely control plant growth.

In conclusion, the symbiotic relationship between GRNs and phytohormones is paramount for the complex orchestration of plant development and morphogenesis. Understanding this intricate dance can offer insights into plant development, crop improvement, and sustainable agriculture practices.

## Author contributions

MA: Conceptualization, Writing – original draft, Writing – review & editing. YZ: Conceptualization, Funding acquisition, Writing – review & editing. WL: Writing – review & editing.
